# Ascending Cholangitis Caused by Methicillin-Resistant Staphylococcus aureus Species in a Patient With Cystic Fibrosis

**DOI:** 10.7759/cureus.17045

**Published:** 2021-08-10

**Authors:** Yasmeen Obeidat, Davinder Singh, Saba AlTarawneh, Joseph Simmons, Adnan Elghezewi, Eva Patton-Tackett, Wesam Frandah

**Affiliations:** 1 Internal Medicine, Marshall University Joan C. Edwards School of Medicine, Huntington, USA; 2 Gastroenterology and Hepatology, Marshall University Joan C. Edwards School of Medicine, Huntington, USA; 3 Internal Medicine/Gastroenterology, Marshall University Joan C. Edwards School of Medicine, Huntington, USA

**Keywords:** methicillin resistant staphylococcus aureus (mrsa), acute cholangitis, rare organism, endoscopic retrograde cholangiopancreatography (ercp), cystic fibrosis liver

## Abstract

Ascending cholangitis is a bacterial infection of the extra-hepatic biliary system and presents as a life-threatening systemic condition. Increased bacterial loads and biliary obstruction favor bacterial translocation into the vascular and lymphatic systems. Common organisms isolated are *Escherichia Coli*, *Klebsiella*, *Enterococcus *species, and *Enterobacter *species*. *Methicillin-resistant *Staphylococcus aureus* (MRSA) is a rare isolate in ascending cholangitis. We present a case of a 24-year-old patient with cystic fibrosis who presented with epigastric abdominal pain, low-grade fever, jaundice, dark urine, and nausea for two days. Initial workup revealed elevated liver enzymes, hyperbilirubinemia, leukocytosis, and an ultrasound which showed common bile duct dilation to 14 mm with choledocholithiasis. He underwent endoscopic retrograde cholangiopancreatography (ERCP) with stone extraction and bile fluid culture. Cultures grew out MRSA and the patient was treated with appropriate antibiotic therapy. The mainstay of therapy for ascending cholangitis is adequate hydration, antibiotics, and biliary decompression. Early recognition of the offending organism is critical in guiding therapy. Current guidelines focus on the empiric treatment of Gram-negative and anaerobic bacteria. Clinicians should be aware of the possibility of less common pathogens (such as MRSA), especially in a patient who is decompensating despite antibiotic therapy.

## Introduction

Ascending cholangitis, also known as acute cholangitis, is a bacterial infection of the extra-hepatic biliary system and presents as a life-threatening systemic condition. Earlier studies have reported a mortality rate between 11% and 27% [1]. The average age of patients with ascending cholangitis is between 50 and 60 years [2]. Biliary obstruction and inflammation favor bacterial translocation into the vascular and lymphatic systems [3]. The most common isolated pathogens found in ascending cholangitis are *Escherichia coli* (25%-50%), *Klebsiella* (15%-20%), *Enterococcus *species (10%-20%), and *Enterobacter* species (5%-10%) [2]. While coliform organisms represent the most common culprits isolated in ascending cholangitis, methicillin-resistant *Staphylococcus aureus* (MRSA) is rarely observed. We highlight a rare case of a young 24-year-old male patient with acute cholangitis with blood cultures positive for MRSA infection.

## Case presentation

Our patient is a 24-year-old-male with a medical history of cystic fibrosis (F508delta-CFTR), gastroesophageal reflux disease (GERD), hiatal hernia post-Nissen fundoplication, choledocholithiasis, and cholecystitis post-cholecystectomy. He presented to the emergency department with sharp, persistent, and non-radiating epigastric pain for two days. It was associated with nausea, vomiting, low-grade fever, jaundice, and dark urine.

His temperature was 100.3 F, the remainder of his vital signs were within normal range. He appeared to be in mild distress on physical examination with epigastric tenderness and scleral icterus. Initial laboratory workup was significant for a white blood cell count of 14.7 x 10^9^ cells per liter, mild hypokalemia with a potassium level of 3.2 mEq/L, elevated liver enzymes (alkaline phosphatase [ALP] 558 U/L, aspartate aminotransferase [AST] 99 U/L, alanine aminotransferase [ALT] 147 U/L), and total bilirubin of 4.4 mg/dL. Urinalysis revealed a large amount of bilirubin, moderate urobilinogen, and trace leukocyte esterase. An abdominal ultrasound revealed heterogeneous liver echogenicity with probable areas of fatty infiltration and intrahepatic bile duct dilatation. The common bile duct (CBD) was measured 14 mm with a stone present within the CBD.

**Figure 1:**
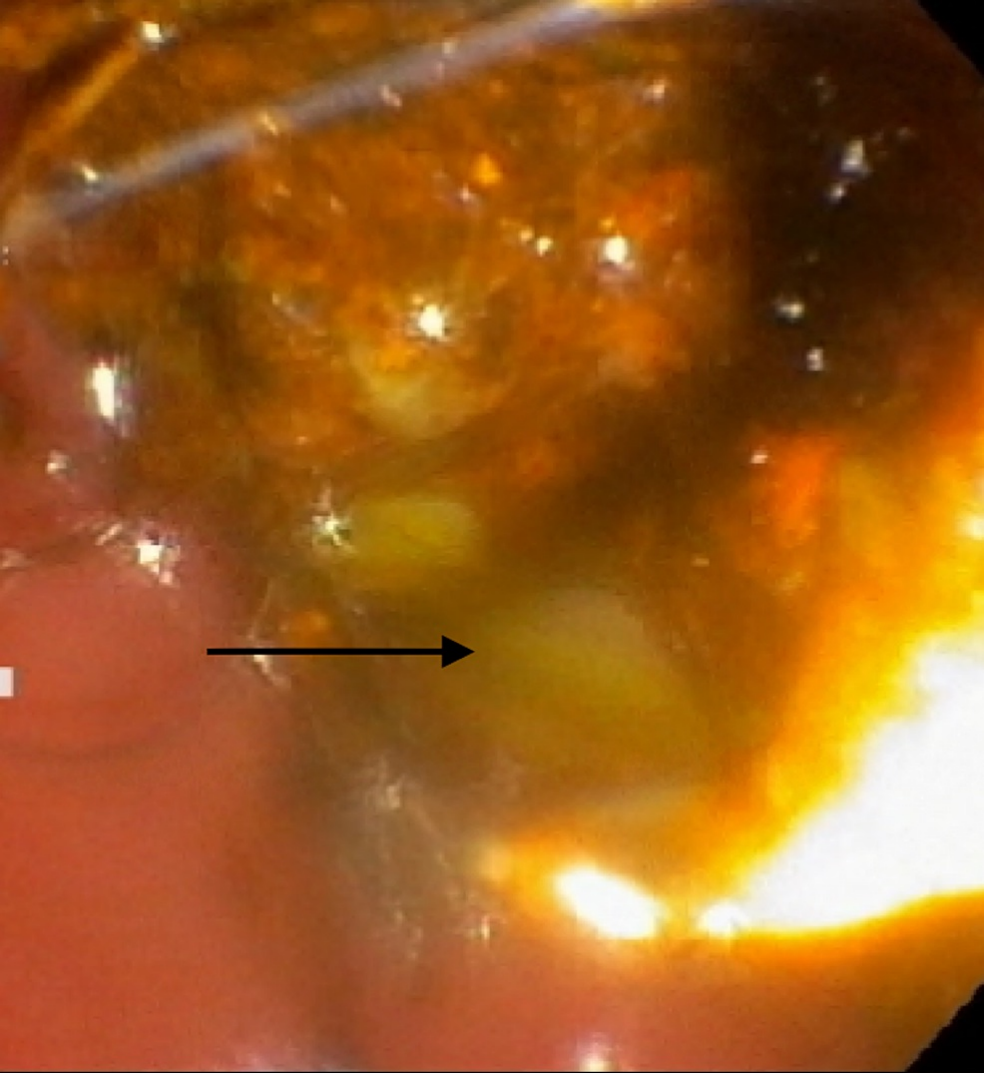
Endoscopic retrograde cholangiopancreatography (ERCP) demonstrating frank pus draining from the major papilla.

**Figure 2:**
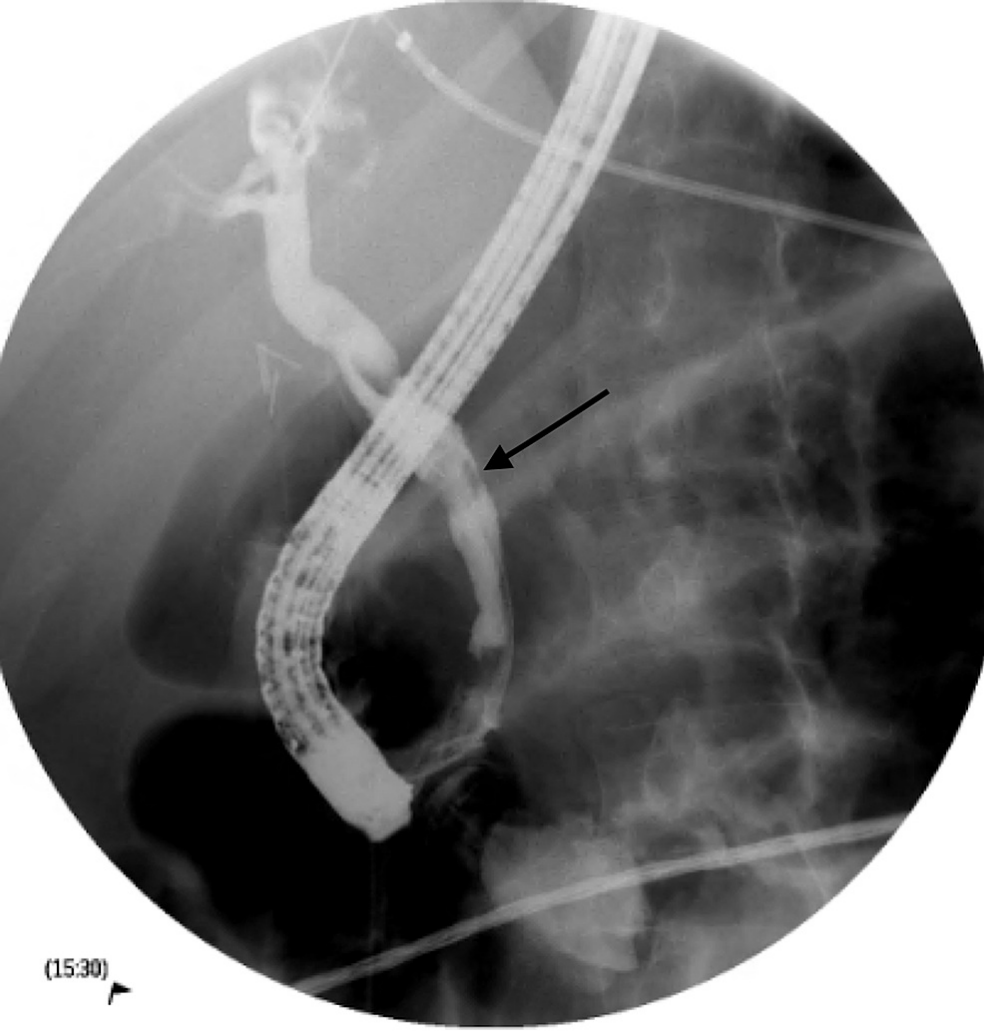
Endoscopic retrograde cholangiopancreatography (ERCP) with fluoroscopy demonstrating filling defects within the proximal to the distal common bile duct.

Broad-spectrum antibiotics including vancomycin and piperacillin-tazobactam were started after obtaining blood, urine, and sputum cultures. Endoscopic retrograde cholangiopancreatography (ERCP) showed dilatation of the biliary tree and stones in the CBD. After a sphincterotomy was performed, purulent drainage and bile were noted (Figures 1, 2). Cultures were collected with the removal of the stones via balloon extraction, and a plastic stent was placed. The patients’ blood and bile cultures revealed MRSA. The patient underwent a transesophageal echocardiogram (TEE) with no evidence of endocarditis. Repeat blood cultures were negative, The patient was discharged home after improvement of his symptoms on intravenous antibiotics.

## Discussion

Ascending cholangitis is a life-threatening condition. Prompt recognition and timely intervention can reduce mortality, morbidity, and reoccurrence. Diagnosis is established with clinical features, imaging studies, and laboratory results. The clinical presentation is correlated with the severity of cholangitis. Classic symptoms include high fever, abdominal pain, and jaundice which is also known as Charcot’s triad [4]. Severe and more alarming symptoms include hypotension and altered mental status is termed Reynold’s pentad [4]. Cases of acute cholangitis can be further broken down into severity. For instance, mild grade I is described as acute cholangitis that responds to initial medical treatment, and moderate grade II is described as acute cholangitis without organ dysfunction that does not respond to initial medical treatment [5]. Lastly, grade III is described as severe cholangitis where patients can present with organ dysfunction, hypotension, and some cases altered mental status [5]. The patient described in the case had moderate grade II severity of acute cholangitis where initial medical management failed.

Initial laboratory workup should include complete blood count, erythrocyte sedimentation rate, complete metabolic profile, hepatic enzymes, and blood cultures. The most commonly isolated pathogens found in ascending cholangitis are *E. coli* (25%-50%), *Klebsiella *(15%-20%), *Enterococcus* species (10%-20%), and *Enterobacter* species (5%-10%) [2]. Another modality used in isolating pathogens is ERCP-obtained bile cultures. The ERCP-obtained bile cultures technique provides a significantly higher yield versus blood cultures [6]. One paper reports that the bile cultures can be positive in 59% to 93% of the acute cholangitis cases [2]. Bacteriologic sampling through blood cultures and bile cultures provides physicians important information needed to correctly tailor the antibiotics based on the isolated organisms.

Imaging modalities often aid recognition of acute cholangitis when clinical suspicion is high. The most common modalities used are ultrasound of the abdomen, computed tomography (CT), and magnetic resonance cholangiopancreatography (MRCP) [7]. Because ascending cholangitis causes high biliary intraductal pressure, biliary secretion of antibiotics is impaired. As a result, ERCP can be both diagnostic and therapeutic by reducing intraductal pressure through the removal of stones and sphincterotomy [8]. ERCP provides an important tool to visualize the biliary ducts. Success rates using ERCP are reported to be 98% and are safer than surgical and percutaneous interventions [9]. The complication rate of ERCPs is reported to be 1.38% with the mortality rate being 0.21% [9]. ERCP is the gold standard therapy and as such clinical and lab improvement is expected.

The treatment for ascending cholangitis involves fluid resuscitation, antibiotics, and biliary decompression. Biliary decompression remains the cornerstone of therapy and should be performed when the patient is not improving due to septicemia [10]. Lack of accurate microbiologic diagnosis and inappropriate antibiotic coverage can lead to septicemia in up to 50% of patients [11]. With regard to bacteremia from ascending cholangitis, organisms include *Klebsiella*, *Proteus*, *Pseudomonas aeruginosa*, and *E. coli* [11]. Less common are those that include Gram-positive bacteria and fungi. MRSA bacteremia from ascending cholangitis is extremely low on the differential. Vancomycin is considered a first-line antibiotic option for MRSA bacteremia with susceptible minimum inhibitory concentrations [12]. Early administration and the correct choice of antibiotics are associated with a reduced incidence of septicemia.

## Conclusions

Ascending cholangitis is a potentially life-threatening infection of the biliary tract. While blood cultures present a quick and easy method to isolate the causative organism, bile cultures obtained during ERCP provide a significantly higher yield and can aid in tailoring antibiotics appropriately. Most of the literature concerning ascending cholangitis recommends coverage for Gram-negative and anaerobic organisms with antibiotics. When traditional antibiotic therapy fails clinicians should have a high index of suspicion for uncommon bacteria such as MRSA in ascending cholangitis.
